# Dermatomyositis with intrahepatic cholangiocarcinoma: a case report and data mining based on machine learning

**DOI:** 10.3389/fonc.2023.1206043

**Published:** 2023-08-18

**Authors:** Xusheng Zhang, Bendong Chen

**Affiliations:** ^1^ Ningxia Medical University, Yinchuan, China; ^2^ General Hospital of Ningxia Medical University, Yinchuan, China

**Keywords:** dermatomyositis, intrahepatic cholangiocarcinoma, paraneoplastic dermatomyositis, PARP12, MT1M

## Abstract

Cancer secondary to dermatomyositis (DM) is defined as paraneoplastic dermatomyositis, which is one of the major subtypes of DM. However, cases of DM with intrahepatic cholangiocarcinoma (ICC) are rarely reported. In the course of our clinical work, we encountered a case of a middle-aged female patient who was diagnosed with DM for 7 years and then diagnosed with ICC, and we would like to share this case. In addition, in order to further investigate the deeper mechanism of ICC associated with DM, we also analyzed the dataset related to DM and ICC in the Gene Expression Omnibus (GEO) database based on the machine learning methods and found that poly(ADP-ribose) polymerase family member 12 (PARP12) and metallothionein 1M (MT1M) were closely associated with ICC secondary to DM. They are potentially important biomarkers for predicting the occurrence of ICC in patients with DM.

## Graphical Abstract

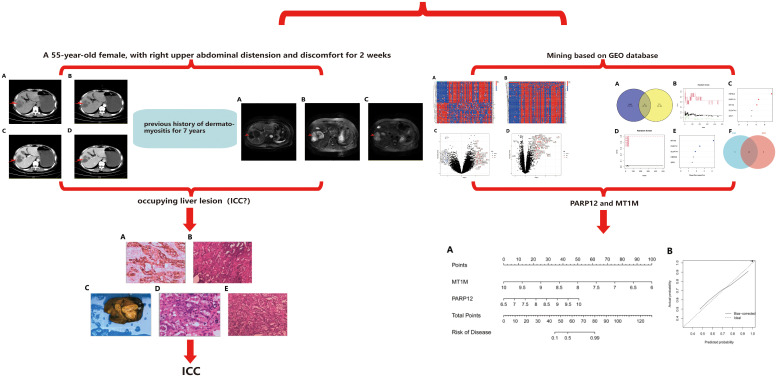

## 1 Research background

Intrahepatic cholangiocarcinoma (ICC) is one of the major pathological types of primary hepatic carcinoma, accounting for approximately 15%–20% of all primary hepatic carcinoma and occurring in the epithelial cells in secondary bile ducts and higher (1). The main features of the disease are high heterogeneity, high invasiveness, and very poor prognosis. According to tumor epidemiological findings, the incidence of ICC has continued to increase in recent years and will remain at a certain increase for a longer period in the future (2). The risk factors of ICC include chronic hepatitis, cirrhosis, biliary inflammatory diseases, sclerosing cholangitis, and intrahepatic bile duct stones. Up to now, surgical treatment is still the preferred treatment modality for ICC. In recent decades, with the continuous refinement of tumors and the development and iteration of medical technology, the treatment of ICC has been refined and diversified. Therapies such as adjuvant chemotherapy, targeted therapy, and immunotherapy have been applied to patients with advanced and metastatic ICC (3–5). However, the treatment of ICC remains very tricky so far, which is related to the anatomical structure and the characteristics of the disease. Some studies show that for patients with resectable cholangiocarcinoma, the 5-year survival rate is approximately 10%–40%, while in unresectable cholangiocarcinoma, the patient’s 3-year annual survival rate is less than 2%, and the 5-year survival rate is approximately 0% (5). The mortality rate of intrahepatic cholangiocarcinoma is on the rise compared to extrahepatic cholangiocarcinoma from 1995 to 2016, which seems to be in contradiction with the development of the level of treatment. This may be related to improvements in disease diagnostic techniques, but further proof is needed (6).

Dermatomyositis (DM) is a main subtype of idiopathic inflammatory myopathy (IIM), which is characterized by multi-system involvement of the skin and muscles, which is more common in women and can develop in both adults and children (7). Studies have shown that the risk of malignancy in DM patients is significantly higher than in the healthy population, with one study mentioning that it is approximately 4.66 times higher than in the normal population (8). The risk of malignancy is highest within 12 months after diagnosis of DM (standardized incidence ratio equaled 17), with the strongest correlation with malignancy in anti-tif1-γ-positive patients, whose cancer risk was shown to be 9.37 times higher than in the normal population (9). As of now, studies have shown that 15%–30% of DM patients suffer from related malignancies (10), and the relatively clear relationship between cancer and DM in current studies includes breast cancer, hematologic malignancies, colorectal cancer, lung cancer, ovarian cancer, prostate cancer, and nasopharyngeal cancer (11–13). Cancer has become one of the important causes of death in patients with DM (14). Up to now, there are few reports of ICC associated with DM.

## 2 Case presentation

Here, we will share a case of ICC associated with DM that we encountered in our clinical work. The patient was a 55-year-old woman who presented to the clinic with “right upper abdominal distension for 2 weeks”. Physical examination revealed no jaundice, flat abdomen, no gastrointestinal type, no peristaltic waves, and no abdominal wall varices. The bowel sounds were three times every minute. The whole abdomen is soft, with no pressure pain and rebound pain, no muscle tension, and no palpable abdominal mass. Her Murphy’s sign was negative. Past medical history indicated that the patient had a previous history of dermatomyositis for 7 years, and the rest was not specific.

## 3 Ancillary examinations

### 3.1 Computed tomography

**Figure 1:**
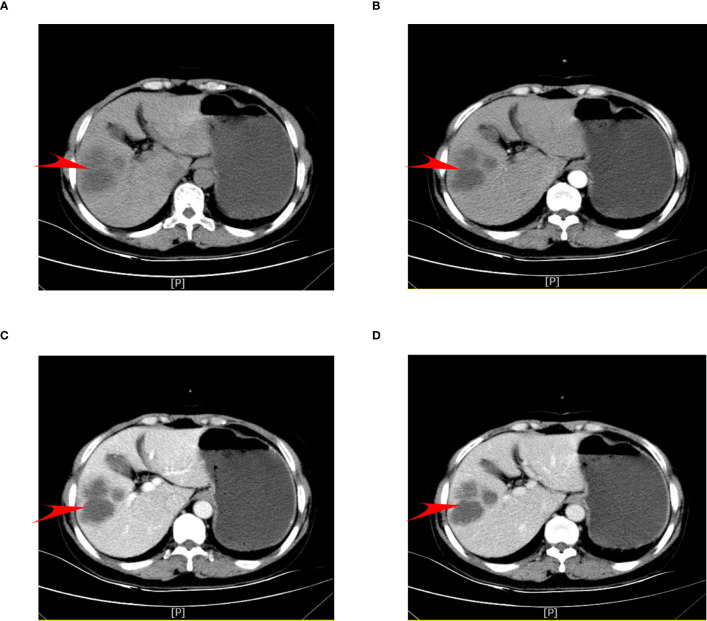
Patient’s preoperative abdominal CT axial scan and enhancement. **(A)** Plain phase. **(B)** Arterial phase. **(C)** Venous phase. **(D)** Delayed phase.

The abdominal computed tomography (CT) axial scan and enhancement suggested hypodense foci in the right posterior lobe of the liver and multiple cysts in the liver (**Figures 1A–D**). Combined with the different periods of CT, it was seen that the occupancy of the right lobe of the liver was obvious, but since the CT presentation of this occupying lesion was not typical, we could not conclude the nature of the occupancy of the patient’s liver simply by CT examination.

### 3.2 MRI scan

**Figure 2:**
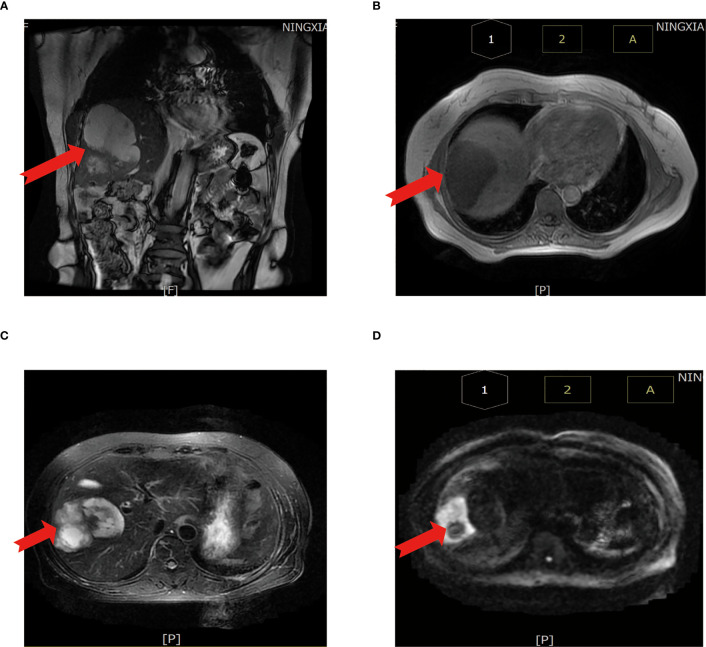
Abdominal MRI scan. **(A)** MRI coronal T2WI sequence. **(B)** T1WI sequence shows a low signal. **(C)** T2WI sequence shows a high signal. **(D)** DWI sequence shows a high signal. The red arrow points to the cancer foci. T1WI, T1-weighted imaging; T2WI, T2-weighted imaging; DWI, diffusion-weighted imaging.|.

Magnetic resonance imaging (MRI) of the abdomen suggests that abnormal signal foci were seen in the right lobe of the liver with a low signal in T1-weighted imaging (T1WI) (**Figure 2A**), high signal in T2-weighted imaging (T2WI) fat suppression (**Figure 2B**), and high signal in diffusion-weighted imaging T2WI sequence (T2WI and DWI) (**Figures 2C, D**), with edge and separation enhancement after enhancement. Multiple round-like foci of long T1 and long T2 signals were seen in the liver, with a diameter of approximately 0.5 cm, and no significant enhancement was seen after the enhancement scan. The diagnosis was abnormal signal foci in the right lobe of the liver, considering the possibility of an abscess and multiple intrahepatic cysts.

### 3.3 Tumor marker assay

Carbohydrate antigen 199 (CA19-9) was 1,000.00 U/ml. Carbohydrate antigen (CEA) assay was 7.02 ng/ml. Carbohydrate antigen 125 (CA12-5) was 183.20 U/ml. Alpha-fetoprotein (AFP) was normal.

## 4 Preliminary diagnosis and treatment

Combined with the patient’s medical history, imaging presentation, and tumor marker levels, the patient was diagnosed with liver-occupying lesions and dermatomyositis. After evaluation, the patient was eligible for surgical resection, and the patient was subsequently treated surgically. The surgical procedure was as follows.

Tracheal intubation was performed after general anesthesia was administered to the patient. The skin, muscle, and peritoneum were incised layer by layer along the right subcostal margin, and the incision was approximately 20 cm long. Exploration of the abdominal cavity did not show any foci of the abdominal cavity, omentum, or abdominal wall displacement. The liver was normal in morphology and soft in texture and showed no sclerosis. The adhesions around the gallbladder were lax, and after the separation of the adhesions, a space-occupying lesion was seen in the right hepatic lobe, which was cystic and soft when it protruded toward the diaphragmatic plane. The lesion protruding to the visceral surface showed solid changes and protruded from the liver surface, partly located behind the gallbladder; the rest of the liver showed no abnormality. Considering that part of the tumor is located behind the gallbladder, the gallbladder cannot be preserved, so we performed “partial hepatectomy and cholecystectomy” on the patient: the right half of the liver was fully freed, the gallbladder triangle was anatomized, and the gallbladder was regularly resected. The hepatoduodenal ligament was dissected; the right and left hepatic ducts, the right and left hepatic arteries, and the right and left branches of the portal vein were isolated; the right branch of the portal vein and the right hepatic artery were blocked. The tumor was completely resected with an ultrasonic knife 2 cm from the edge of the tumor, and the bile ducts and blood vessels encountered in the cross-section were exactly ligated or sutured and blocked for approximately 20 minutes. The patient bled approximately 300 ml during the operation.

## 5 Pathology and immunohistochemical results

### 5.1 Intraoperative frozen specimen paraffin section report

**Figure 3:**
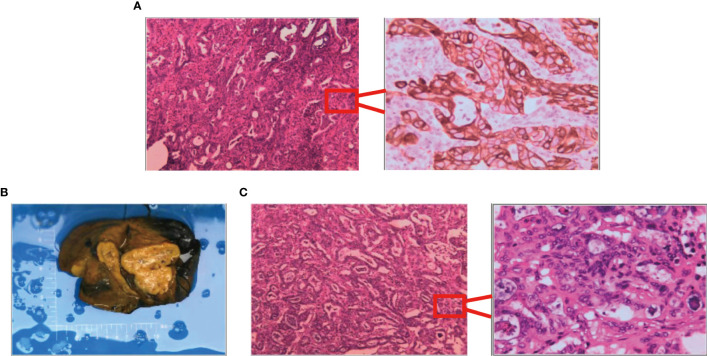
Results of surgically resected pathological tissue sent for examination. **(A)** Intraoperative frozen pathological sections. **(B)** Surgical resection of the whole specimen; the volume of cancer foci was approximately 6 cm × 5 cm × 4 cm. **(C)** Postoperative pathological specimens examined.

First evaluation: (liver) pathological examination of the sample sampling suggested that no cancerous tissue was seen. Second delivery: moderately differentiated adenocarcinoma of intrahepatic bile duct with vascular and nerve invasion was seen (**Figure 3A**).

### 5.2 Postoperative pathological specimen sent for examination report

A liver cyst combined with moderately differentiated adenocarcinoma of the intrahepatic bile duct was seen, and the cancer tissue invaded the liver envelope (**Figures 3B, C**).

Immunohistochemical results were as follows: CKpan (+), CK7 (+), CK20 (−), CDX-2 (−), Ki67 (index 80%), Vimentin (−), TTF-1 (−), Tg (−), CA125 (+), Pax-8 (−), CA199 (+), GATA-3 (−), Villin (+) CR (+), MC (−), D2-40 (−), and CEA (+).

## 6 Final diagnosis and recovery of the patient’s condition

Combined with the pathological diagnosis and immunohistochemical results, the patient was finally diagnosed with ICC intermediate differentiated adenocarcinoma. The patient made a good overall recovery after surgery and expressed satisfaction with the surgical treatment. After surgery, she was transferred to the Department of Medical Oncology to continue chemotherapy and DM-related treatment. Up to our last follow-up visit, the patient showed no signs of cancer recurrence.

Based on this case report, the deep-seated association between the two diseases was of deep interest to us, so we further explored the relationship between the two diseases at the genetic level. We searched the Gene Expression Omnibus (GEO) database and found DM datasets GSE46239/GSE1551 and the ICC dataset GSE32225. Then, based on machine learning methods, we explored the three datasets to identify the causes of ICC associated with DM.

## 7 Results

### 7.1 Differential expression analysis

**Figure 4:**
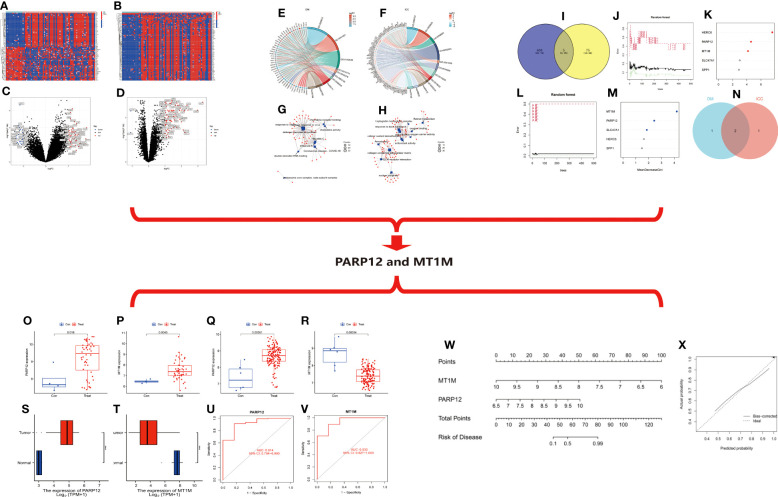
**(A)** Heat map of differentially expressed genes in ICC. **(B)** Heat map of differential gene expression in DM. **(C)** Volcano map of differentially expressed genes in ICC. **(D)** Volcano map of differentially expressed genes in DM. **(E)** Circle diagram of GO/KEGG enrichment analysis of differentially expressed genes in DM. **(F)** Circle diagram of GO/KEGG enrichment analysis of differentially expressed genes in ICC. **(G)** Network diagram of enrichment analysis of differentially expressed genes in DM. **(H)** Network diagram of enrichment analysis of differentially expressed genes in ICC. (**I–N**) Random forest tree-based screening for signature genes. **(I)** Intersection Wayne plot of differentially expressed genes. **(G, K)** Random forest tree. **(G)** DM. **(K)** ICC. Green: error of cross-validation in the normal group. Red: error of cross-validation in the disease group. Black: error of cross-validation in the overall sample. **(L, M)** Circle plot of gene importance scores based on random forest model. **(L)** DM. (**M**) ICC. **(N)** Venn diagram of the intersection of genes with top three importance scores in DM and ICC. **(O–V)** Expression and diagnostic efficacy analysis of characteristic genes. **(O, P)** Expression analysis of PARP12 and MT1M in DM. (**Q, I**) Expression analysis of PARP12 and MT1M in ICC. **(S, T)** Expression level validation of PARP12 and MT1M in ICC. **(U, V)** Diagnostic efficacy analysis of PARP12 and MT1M in ICC. Con, normal; Treat, DM or ICC. **(W, X)** Nomogram risk model construction. **(W)** Columnar graph model. **(X)** Calibration curve. Con, normal; Treat, ICC. ICC, intrahepatic cholangiocarcinoma; DM, dermatomyositis; GO, Gene Ontology; KEGG, Kyoto Encyclopedia of Genes and Genomes.

Based on the DM and ICC dataset, the differentially expressed genes in the two diseases were screened with the screening condition of |LogFC| ≥ 1, p < 0.05. The results showed that there were 635 differentially expressed genes in ICC and 80 differentially expressed genes in DM, and the heat map (**Figures 4A, B**) and volcano map (**Figures 4C, D**) of the differentially expressed genes in the two diseases were plotted.

### 7.2 GO/KEGG enrichment analysis

We then performed functional and pathway enrichment analyses of the differentially expressed genes in two diseases. The results showed that the differentially expressed genes in DM were mainly enriched in response to the virus, defense response to the virus, and defense response to symbiont at the biological process (BP) level. The molecular function (MF) level is mainly enriched in double-stranded RNA binding, chemokine receptor binding, and chemokine activity and mainly enriched in coronavirus disease–COVID-19, influenza A, hepatitis C, and other pathways (**Figures 4E, G**). ICC differential genes at the BP level are mainly enriched in response to a toxic substance, detoxification, and cellular oxidant detoxification. At the level of cellular component (CC), they are mainly enriched in the collagen-containing extracellular matrix, nuclear periphery, etc. At the level of MF, they are mainly enriched in antioxidant activity, oxygen binding, etc. The pathways are mainly enriched in extracellular matrix (ECM)–receptor interaction, retinoid metabolism, etc. (**Figures 4F, H**).

### 7.3 Screening of signature genes based on random forest trees

Then, we took the intersection of the differentially expressed genes in the two diseases, and the results showed that there were five common differentially expressed genes in the two diseases: MT1M, HERC6, SPP1, PARP12, and SLC47A1 (**Figure 4I**). Next, we ranked the importance of these five intersecting differential genes in the two diseases based on the random forest (RF) tree algorithm; the best NTree was selected based on the minimum cross-validation error in 10 cross-validations. The MTRY and NTree were set to 139 and 500, respectively, in DM, and 19 and 500, respectively, in ICC (**Figures 4J–M**). The top three genes in the importance ranking were further screened for cross-validation, resulting in the final two differentially expressed genes, namely, poly(ADP-ribose) polymerase family member 12 (PARP12) and metallothionein 1M (MT1M) (**Figure 4N**).

### 7.4 Expression of characteristic genes and analysis of diagnostic efficacy

We then analyzed the expression of PARP12 and MT1M in DM and ICC. The results showed that PARP12 and MT1M were both significantly upregulated in DM (**Figures 4O, P**). In contrast, PARP12 expression was upregulated in ICC, while MT1M expression levels were downregulated in ICC (**Figures 4Q, R**). The same results were obtained using the cholangiocarcinoma data from The Cancer Genome Atlas (TCGA) database for validation (**Figures 4S, T**). We also analyzed the diagnostic efficacy of PARP12 and MT1M for ICC, and the results showed that their diagnostic receiver operating characteristic–area under the curve (ROC-AUC) was 0.914 and 0.933, respectively, which both showed good diagnostic ability for ICC (**Figures 4U, V**).

### 7.5 Nomogram risk model construction

Then, we constructed a risk prediction model for ICC based on the expression levels of PARP12 and MT1M in ICC (**Figure 4W**). From the nomogram risk model, it was seen that the decreased expression level of MT1M was a risk factor for ICC, and the upregulated expression of PARP12 was also a risk factor for ICC. We then evaluated the predictive efficacy of this risk model using a calibration curve (**Figure 4X**), and the results showed a good fit of the calibration curve, suggesting that the risk model has good predictive efficacy.

## 8 Discussion

The patient presented to the clinic with right upper abdominal distension and discomfort, and after completing abdominal imaging, we considered cancer as a possibility; however, the results of the imaging report do not indicate it clearly. The patient’s tumor markers showed a significant increase in the expression of CA19-9, CEA, and CA12-5. It was suggested that the occupying lesion might be a malignant tumor. During the history-taking process, we learned that the patient had been diagnosed with DM for 7 years and was taking regular medication. The patient was finally diagnosed with intrahepatic cholangiocarcinoma, a moderately differentiated adenocarcinoma, in combination with the intraoperative pathological specimen frozen for examination, postoperative pathological examination report, and immunohistochemical results.

Paraneoplastic dermatomyositis is more common in clinical practice and is one of the important subtypes of DM, some of the cancer types associated with DM have been studied in depth, and some relevant antibodies have been proposed to be associated with the development of the paraneoplastic syndrome. However, cases of DM with ICC have been very rare so far, and only three cases were found through a literature search. In a case reported by Knowles BP et al. in 2006, a 38-year-old Ethiopian man presented with DM, and ultrasound and CT scan of the patient’s abdomen showed a heterogeneously enhancing 4-cm hypodense mass adjacent to the gallbladder in segment 4B of the liver, for which a hepatic wedge resection + cholecystectomy + hilar lymph node dissection was performed. The pathological histological analysis finally proved that the tumor was a hypofractionated intrahepatic cholangiocarcinoma with hilar lymph node metastasis (15). In 2016, Koung Jin Suh et al. reported on a patient with DM with paraneoplastic dermatomyositis diagnosed *via* [^18^F]-fluorodeoxyglucose (FDG) positron emission tomography–computed tomography (PET-CT) scan. Because cancer cells are generally more metabolically active than normal tissues, they require more energy and therefore absorb and retain more energy than non-cancerous cells. Cancer cells take up and retain more glucose. This case presents a patient with DM showing intense proximal muscle diffuse FDG uptake on whole-body PET-CT scans before the development of any muscle symptoms (16). A recently reported case of DM with ICC was identified clinically by Jungo Yasuda et al. in 2018 in a 44-year-old woman suspected of having the paraneoplastic syndrome, who was found to have ICC at the same time during the exacerbation of DM, and the authors concluded that the patient’s paraneoplastic syndrome contributed to the deterioration of DM. The patient was treated surgically and with postoperative chemotherapy after the diagnosis of ICC. The patient was discharged 23 days after surgery, and at the time of discharge, the patient was assessed to have reduced DM, and the treatment was effective (17).

The pathogenesis of DM with malignancy has not been investigated so far, and there are still very few studies in this area. Some of the available studies have hypothesized that the occurrence of the paraneoplastic syndrome may be related to autoimmune disorders, TIF1 gene mutations and heterozygous deletions, reduced immune surveillance, drugs, infections, vitamin D deficiency, and ultraviolet radiation (18–20). Compared with those of breast cancer, colorectal cancer, and lung cancer, the cases of ICC associated with DM have been reported more rarely so far, but the cases of ICC associated with DM inevitably occur in clinical practice. Hence, more studies on ICC associated with DM are needed to provide clinical supporting evidence for its diagnosis and management.

PARP12 is a member of the poly-ADP-ribosyl polymerase (PARP) family (21), which uses NAD+ as a substrate to modify target proteins by attaching ADP-ribose, and is associated with cellular stress responses related to DNA repair. PARPase inhibitors are in clinical trials as tumor therapy and are defined as an interferon-stimulated gene (22, 23). It has been mentioned that PARP12 deficiency increases the migration and invasion of hepatocellular carcinoma (HCC) cells by regulating the epithelial–mesenchymal transition process (24), while PARP12 also seems to have an important potential role in cellular defense against viral infection [24].

MT1M, a member of the metallothioneins, has a high content of cysteine residues that bind various heavy metals. These genes are transcriptionally regulated by both heavy metals and glucocorticoids. MT1M is also highly involved in cancer; for example, in esophageal squamous cell carcinoma (ESCC), MT1M expression levels were found to be downregulated, and MT1M was found to inhibit esophageal carcinoma cell carcinogenesis through the inhibition of epithelial–mesenchymal transition and SOD1/PI3K axis (25). In lung adenocarcinoma, MT1M overexpression was also found to inhibit A549 cell viability and migration ability *in vitro*, reduce MT1M expression, and promote tumor cell proliferation and migration (26). In another study, it was also mentioned that MT1M expression was often downregulated in HCC, which may be associated with methylation of the promoter region and upregulation of miR-545-3p, leading to downregulation of MT1M, which in turn regulates the proliferation, invasion, and migration of hepatocellular carcinoma cells (27, 28).

Next, based on machine learning, we found that PARP12 was significantly upregulated in both DM and ICC, and PARP12 was also found to be a risk factor for ICC in the nomogram risk model, thus inferring that PARP12 may play a role in promoting the development of ICC in DM, while MT1M was upregulated in DM and significantly downregulated in ICC. It is suggested that MT1M expression is a risk factor for ICC, suggesting that we can largely predict the occurrence of ICC by measuring the expression level of MT1M in DM patients, which has a predictive effect on the development of ICC in DM patients. Both of them are potential predictive targets for ICC in DM.

## 9 Conclusion

PARP12 and MT1M are important predictive targets for ICC associated with DM.

## Data availability statement

The original contributions presented in the study are included in the article/**Supplementary Material**. Further inquiries can be directed to the corresponding author.

## Ethics statement

Written informed consent was obtained from the individual(s), and minor(s)’ legal guardian/next of kin, for the publication of any potentially identifiable images or data included in this article.

## Author contributions

Two authors contributed equally to this report, sharing the first authorship. The data collection and analysis were performed jointly. XZ wrote the article, and BC reviewed and revised it. All authors contributed to the article and approved the submitted version.

## References

[1] Banales JM Marin J Lamarca A Rodrigues PM Khan SA Roberts LR . Cholangiocarcinoma 2020: the next horizon in mechanisms and management. Nat Rev Gastroenterol Hepatol (2020) 17:557–88. doi: 10.1038/S41575-020-0310-Z PMC7447603 32606456

[2] Sung H Ferlay J Siegel RL Laversanne M Soerjomataram I Jemal A . Global cancer statistics 2020: globocan estimates of incidence and mortality worldwide for 36 cancers in 185 countries. CA Cancer J Clin (2021) 71:209–49. doi: 10.3322/Caac.21660 33538338

[3] [Standardization for diagnosis and treatment of hepatocellular carcinoma (2022 edition)]. Zhonghua Gan Zang Bing Za Zhi (2022) 30:367–88. doi: 10.3760/Cma.J.Cn501113-20220413-00193 PMC12769260 35545562

[4] Wang M Chen Z Guo P Wang Y Chen G . Therapy for advanced cholangiocarcinoma: current knowledge and future potential. J Cell Mol Med (2021) 25:618–28. doi: 10.1111/Jcmm.16151 PMC7812297 33277810

[5] Blechacz B . Cholangiocarcinoma: current knowledge and new developments. Gut Liver. (2017) 11:13–26. doi: 10.5009/Gnl15568 27928095 PMC5221857

[6] Bertuccio P Malvezzi M Carioli G Hashim D Boffetta P El-Serag HB . Global trends in mortality from intrahepatic and extrahepatic cholangiocarcinoma. J Hepatol (2019) 71:104–14. doi: 10.1016/J.Jhep.2019.03.013 30910538

[7] Patil A Lu J Kassir M Babaei M Goldust M . Adult and juvenile dermatomyositis treatment. J Cosmet Dermatol (2023) 22:395–401. doi: 10.1111/Jocd.15363 36065712

[8] Qiang JK Kim WB Baibergenova A Alhusayen R . Risk of malignancy in dermatomyositis and polymyositis. J Cutan Med Surg (2017) 21:131–6. doi: 10.1177/1203475416665601 27534779

[9] Marzecka M Niemczyk A Rudnicka L . Autoantibody markers of increased risk of malignancy in patients with dermatomyositis. Clin Rev Allergy Immunol (2022) 63:289–96. doi: 10.1007/S12016-022-08922-4 PMC9464248 35147864

[10] Pruessmann W Kleinheinz A Zillikens D Rose C . Histopathological risk factors for malignancy in dermatomyositis. Histopathology (2022) 81:529–35. doi: 10.1111/His.14727 35876260

[11] Leatham H SChadt C Chisolm S Fretwell D Chung L Jp C . Evidence supports blind screening for internal malignancy in dermatomyositis: data from 2 large us dermatology cohorts. Med (Baltimore). (2018) 97:E9639. doi: 10.1097/Md.0000000000009639 PMC5943873 29480875

[12] Hsu JL Liao MF Chu CC Kuo HC Lyu RK Chang HS . Reappraisal of the incidence, various types and risk factors of malignancies in patients with dermatomyositis and polymyositis in Taiwan. Sci Rep (2021) 11:4545. doi: 10.1038/S41598-021-83729-5 33633147 PMC7907377

[13] Teoh JW Yunus RM Hassan F Ghazali N Abidin ZA . Nasopharyngeal carcinoma in dermatomyositis patients: A 10-year retrospective review in hospital Selayang, Malaysia. Rep Pract Oncol Radiother. (2014) 19:332–6. doi: 10.1016/J.Rpor.2014.02.005 PMC4150095 25184058

[14] Marie I . Morbidity and mortality in adult polymyositis and dermatomyositis. Curr Rheumatol Rep (2012) 14:275–85. doi: 10.1007/S11926-012-0249-3 22410829

[15] Knowles BP Corcoran NM Usatoff V . Reading the signs: occult metastatic cholangiocarcinoma detected by full-body screening in dermatomyositis. Anz J Surg (2007) 77:1026–7. doi: 10.1111/J.1445-2197.2007.04307.X 17931277

[16] Suh KJ Park JK Cho S Park H Baek HW Lee K . Dermatomyositis in A patient with cholangiocarcinoma detected by an [(18)F]-fluorodeoxyglucose positron emission tomography-computed tomography scan. Cancer Res Treat (2016) 48:848–52. doi: 10.4143/Crt.2014.310 PMC4843747 25797574

[17] Yasuda J Onda S Shiozaki H Gocho T Shiba H Yanaga K . A successfully treated case of intrahepatic cholangiocarcinoma with exacerbation of dermatomyositis. Case Rep Gastroenterol (2018) 12:622–8. doi: 10.1159/000493185 PMC6244101 30483040

[18] Tiniakou E Mammen AL . Idiopathic inflammatory myopathies and malignancy: A comprehensive review. Clin Rev Allergy Immunol (2017) 52:20–33. doi: 10.1007/S12016-015-8511-X 26429706

[19] Pinal-Fernandez I Ferrer-Fabregas B Trallero-Araguas E Balada E Martinez MA Milisenda JC . Tumour tif1 mutations and loss of heterozygosity related to cancer-associated myositis. Rheumatol (Oxford). (2018) 57:388–96. doi: 10.1093/Rheumatology/Kex413 PMC5850766 29149307

[20] Thompson C Piguet V Choy E . The pathogenesis of dermatomyositis. Br J Dermatol (2018) 179:1256–62. doi: 10.1111/Bjd.15607 28542733

[21] Hu F Li C Ye Y Lu X Alimujiang M Bai N . Parp12 is required for mitochondrial function maintenance in thermogenic adipocytes. Adipocyte (2022) 11:379–88. doi: 10.1080/21623945.2022.2091206 PMC9351573 35916471

[22] Liu SY Sanchez DJ Aliyari R Lu S Cheng G . Systematic identification of type I and type Ii interferon-induced antiviral factors. Proc Natl Acad Sci U S A. (2012) 109:4239–44. doi: 10.1073/Pnas.1114981109 PMC3306696 22371602

[23] Welsby I Hutin D Gueydan C Kruys V Rongvaux A Leo O . Parp12, an interferon-stimulated gene involved in the control of protein translation and inflammation. J Biol Chem (2014) 289:26642–57. doi: 10.1074/Jbc.M114.589515 PMC4176246 25086041

[24] Shao C Qiu Y Liu J Feng H Shen S Saiyin H . Parp12 (Artd12) suppresses hepatocellular carcinoma metastasis through interacting with fhl2 and regulating its stability. Cell Death Dis (2018) 9:856. doi: 10.1038/S41419-018-0906-1 30154409 PMC6113207

[25] Li D Peng W Wu B Liu H Zhang R Zhou R . Metallothionein mt1m suppresses carcinogenesis of esophageal carcinoma cells through inhibition of the epithelial-mesenchymal transition and the Sod1/Pi3k axis. Mol Cells (2021) 44:267–78. doi: 10.14348/Molcells.2021.2179 PMC8112171 33820882

[26] Xu W Jiang GJ Shi GZ Chen MZ Ma TL Tan YF . Metallothionein 1m (Mt1m) inhibits lung adenocarcinoma cell viability, migration, and expression of cell mobility-related proteins through Mdm2/P53/Mt1m signaling. Transl Cancer Res (2020) 9:2710–20. doi: 10.21037/Tcr.2020.02.61 PMC8798298 35117630

[27] Changjun L Feizhou H Dezhen P Zhao L Xianhai M . Mir-545-3p/Mt1m axis regulates cell proliferation, invasion and migration in hepatocellular carcinoma. BioMed Pharmacother. (2018) 108:347–54. doi: 10.1016/J.Biopha.2018.09.009 30227328

[28] Fu CL Pan B Pan JH Gan MF . Metallothionein 1m suppresses tumorigenesis in hepatocellular carcinoma. Oncotarget (2017) 8:33037–46. doi: 10.18632/Oncotarget.16521 PMC5464848 28380433

